# College students’ learning perceptions and outcomes in different classroom environments: A community of inquiry perspective

**DOI:** 10.3389/fpsyg.2022.1047027

**Published:** 2022-12-01

**Authors:** Yan Hu, Jinyan Huang, Fanzhe Kong

**Affiliations:** ^1^School of Education, South-Central Minzu University, Wuhan, Hubei, China; ^2^School of Teacher Education, Jiangsu University, Zhenjiang, China

**Keywords:** smart classroom, “quasi smart” classroom, learning perceptions, learning outcomes, community of inquiry (CoI) framework

## Abstract

**Introduction:**

This study examined the significant differences in Chinese college students’ learning perceptions and outcomes between the “quasi smart” and smart classrooms under the perspective of the community of inquiry (CoI) framework.

**Methods:**

The participants were 275 freshmen students who took the “college physics” hybrid course in the spring of 2022 at a four-year university in central China. Data were collected from the CoI survey, a follow-up focus group interview with ten randomly selected student participants, and a semi-structured interview with the instructor.

**Results and discussion:**

The results indicated that the students’ perceptions of the teaching, social, and cognitive presences were significantly higher in the smart classroom than in the “quasi smart” classroom; further, students in the smart classroom achieved significantly higher course marks than those in the “quasi smart” classroom. The pros and cons of these two different classroom environments were identified by the participating students and their course instructor.

## Introduction

The classroom environment plays an important role in creating the best educational experience for students (Benedict and Hoag, 2004; Stronge, 2007; Vermette, 2009). Different classroom environments provide students with different learning experience (Stronge, 2007). The classroom environment can positively and negatively affect students’ ability to learn to their full potential (Earthman and Lemasters, 2009).

The smart and traditional multimedia classrooms are two different learning environments (Guo and Zheng, 2012; Liu and Zhen, 2019; Wang et al., 2022). The smart classroom refers to a physical classroom integrated with advanced forms of educational technology such as interactive boards, management systems, audio/visual elements, and mobile computing to increase the instructors’ ability and facilitate students’ learning beyond the possibilities of the traditional multimedia classrooms (Wang et al., 2022). Although the traditional multimedia classrooms use multimedia educational technology, they urgently need to be optimized and transformed due to the outdated design concept, obsolete equipments, and a single application mode (Guo and Zheng, 2012). However, it is impossible to switch a traditional multimedia classroom to a smart classroom on a large scale due to various reasons such as budget shortage, a “quasi smart” classroom becomes an alternative, which upgrades the infrastructure of the network, updates the computer and projection display systems, combines the approach of BYOD (bringing your own devices) and cloud desktop, and expands the existing functions of a traditional multimedia classroom (Liu and Zhen, 2019), except that student desks are still fixed and there are steps between the main instructor desk and student desks. Additionally, there is only one display screen in a “quasi smart” classroom but multiple screens in a smart classroom.

The disadvantages of the traditional multimedia classrooms were overcome to some extent in the “quasi smart” classrooms; and some key advantages of the smart classrooms were also introduced into the “quasi smart” classrooms (Liu and Zhen, 2019). The “quasi smart” classrooms are popular in Chinese higher education; and most Chinese university classrooms are “quasi smart” classrooms. The impact of the “quasi smart” classroom in contrast to the smart classroom learning environment on students’ learning perceptions and further on their learning outcomes need to be investigated. Such investigations would yield benefits for both students and their instructors in Chinese universities.

## Literature review

### Research related to smart classrooms and “quasi-smart” classrooms

#### The function of smart classrooms

A typical smart classroom always incorporate digital cameras, recording or casting equipments, multiple interactive whiteboards or touch screen televisions, mobile devices (e.g., tablets and/or smart phones), the wireless Internet, and the educational management software (Kwet and Prinsloo, 2020; Kaur and Bhatia, 2022). Such education technologies could facilitate the content presentation, the management of the classroom, and the access to the learning resources; and they also implement the learning analytics and provide students with real-time feedback (MacLeod et al., 2018). In the smart classroom, the instructors are more capable of providing their students with immediate feedback, and guiding, facilitating, monitoring, and assessing their students’ learning (Li et al., 2021; Lu et al., 2021).

#### Smart classrooms vs. “quasi-smart” classrooms

Previous research has indicated that smart classrooms provide opportunities for students to use digital learning resources and tools in their self-regulated learning and collaborative inquiry learning activities (Zhang et al., 2019). Obviously, instructors could obtain more support for trying the new pedagogies with mobile terminals in smart classrooms (Zhan et al., 2021); and students demonstrated relatively stronger desire for interaction and tendency for utilizing the technology to enhance their learning in the smart classrooms (Cheung and Wang, 2021). For example, Mimouni (2022) found that students were more engaged in learning by using clickers for interaction; and group interaction was also improved, with the pedagogy being changed from teacher-centered to student-centered.

In addition, Sun and Lin (2022) found that the interaction among the learners could be facilitated; learners’ engagement could be enhanced; and their learning motivation could be improved with the classroom response system. Therefore, students would have better perceptions of the smart classroom, interact more in the smart classroom, and ultimately, achieve better performance in the smart classroom in contrast to the “quasi smart” classroom (Xu et al., 2018). Although Yu et al. (2022) reported no significant difference in either the interpersonal interaction or the human–technology interaction between the smart and “quasi smart” classroom environments, students were significantly more engaged in the smart classroom than in the “quasi smart” classroom.

To sum up, research on the effect of classroom settings on teacher-student interaction indicated that students’ autonomy could be more strengthened in the smart classroom than the “quasi smart” classroom; further, students would be more active in face-to-face communication and more likely to collaborate with each other in the smart classroom than the “quasi smart” classroom.

#### Students’ preferences for the smart classroom and its impact on their learning

Research also indicated that students’ preferences for the learning environments could affect their learning outcomes (Fraser, 1998; [Bibr B20][Bibr B57]; Lu et al., 2021; Yu et al., 2022). Students’ preferences for smart classroom indicated their attitude towards specific technology tools and digital learning resources in a smart learning environment (Zhai et al., 2016; Zhang et al., 2020; Yu et al., 2022). Students’ preferences for the smart classroom could improve the effectiveness of the learning environment (MacLeod et al., 2018; Yang et al., 2018). Furthermore, the smart classroom environment could impact students’ higher-order thinking; it was recommended that instructors in the smart classroom focus on improving peer interaction and learning motivation, as well as the smart classroom preferences and learning strategies for students’ higher-order thinking skills (Lu et al., 2021).

#### The reason for smart classrooms being criticized

The rapid development of educational technology has made the smart classroom more social, interactive, flexible, and a student-centered learning environment (Kaufmann and Vallade, 2020; Li and Wong, 2021). Given their advantages, many countries have invested significantly in smart classrooms to promote students’ learning (Temdee, 2021). However, evidence showing that the use of smart classrooms has not met the expectations in promoting students’ learning and academic performance (Mao et al., 2018). Furthermore, smart classrooms have been criticized due to the fact that large amount of money has been spent to build up the most advanced facilities without considering student perspectives (Li et al., 2016; Ze and Fu, 2020; Li and Wong, 2021). Therefore, the role of technology in supporting learning and teaching in the smart classroom needs to be further explored.

### The community of inquiry framework and related research

#### The community of inquiry framework

**FIGURE 1:**
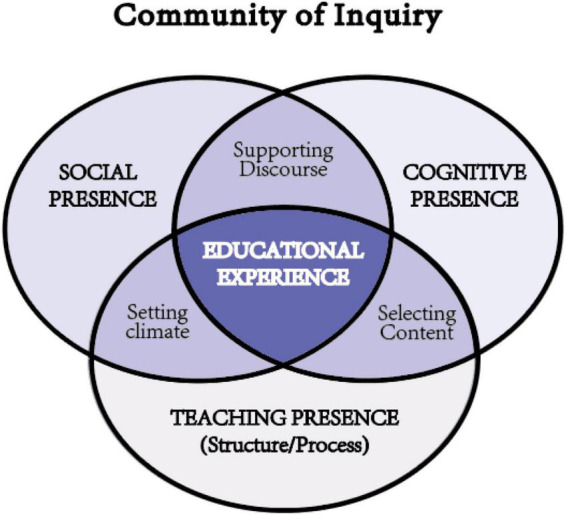
The community of inquiry (CoI) framework. *Permission was granted by Garrison.*

The community of inquiry (CoI) framework to structure the process of learning in an online environment was proposed by Garrison et al. (2000). Garrison (2017) further asserted that “managing and monitoring the dynamic process in thinking deeply and learning collaboratively is the core function of this framework” (p. 24). As shown in Figure 1, three unique and interrelating elements such as cognitive, teaching, and social presences were included in the framework (Garrison et al., 2000). The process of learning is described by the cognitive presence, which is consisted of a triggering event, exploration, integration, and resolution, and the latter phase represents a more advanced cognitive level than the former phase (Garrison et al., 2001). The moderation and guidance of the inquiry is outlined by the teaching presence, which consists of the instructional design and organization, the facilitating discourse, and the direct instruction. The human experience of learning is described by the social presence which is involved affective communication, open communication, and community cohesion (Garrison et al., 2000).

The CoI framework suggests that the profound and meaningful online learning is caused by the interaction of the teaching, social, and cognitive presences perceived by students in the online learning community (Garrison et al., 2000). It provided a coherent framework for understanding e-learning, which has given direction and guidance to educators to facilitate critical discourse and higher order learning (Garrison et al., 2010; Akyol and Garrison, 2011; Garrison and Anderson, 2011; Richardson et al., 2017; Castellanos-Reyes, 2020; Fiock, 2020). The CoI framework provides unique perspectives, methods, and tools for online learning (Garrison et al., 2010; Shea and Bidjerano, 2013; Castellanos-Reyes, 2020). It helps address the challenges and issues of assessing and managing the learning process and outcomes(e.g., Garrison et al., 2010; Garrison and Anderson, 2011; Castellanos-Reyes, 2020).

#### The community of inquiry survey and related research

Since the framework was proposed, it had been largely used, discussed, and examined by researchers in the field (e.g., Garrison and Arbaugh, 2007; Garrison and Anderson, 2011; Garrison, 2017; Castellanos-Reyes, 2020). Arbaugh et al. (2008) developed a 34-item CoI survey about the cognitive, teaching, and social presences, which was proved to be a valid and reliable measuring instrument (Ma et al., 2017). Garrison (2017) commented that the CoI survey advanced significantly the CoI research by effective data analysis and massive studies across disciplines and institutions. Using the CoI survey, some researchers investigated each of the three presences and their relationships within the framework, as well as their relationships with other variables. For example, the causal relationships among the presences of the CoI framework were reported, i.e., the teaching and social presences have a significant influence on the cognitive presence (Shea and Bidjerano, 2009; Garrison et al., 2010; Lin et al., 2015). Furthermore, positive correlations among the teaching, cognitive, and social presences, and student-perceived learning satisfaction were found in online courses (Akyol and Garrison, 2008; Kozan and Richardson, 2014; Patwardhan et al., 2020). Zhan and Hu (2013) posited that online students need higher level social presence, which has a strong effect on their learning achievement and satisfaction, compared to students in the face-to-face classrooms. Within blended learning context, the teaching presence has also direct positive impact on the cognitive and social presences, and indirect positive impact on the learning performance (Law et al., 2019).

#### The community of inquiry framework guiding course designs and related research findings

Some instructors used the framework to guide course designs and make educational decisions (e.g., Garrison et al., 2010; Kovanovi et al., 2019; Fiock, 2020; Patwardhan et al., 2020; Guo et al., 2021). Fiock (2020) structured instructional activities into three presence categories of CoI for instructors use in order to build a community of inquiry into an online course. Garrison et al. (2010) posited that whether or not learners were achieving high levels of critical thinking in online discussion boards depended on the course design. Patwardhan et al. (2020) found that the teaching presence was the primary determinant of learner satisfaction, and course design mediated the relationship between the teaching, social, cognitive presences of the CoI and the learner satisfaction. The research on how the social presence in online project-based learning were related to students’ academic performance showed that the affective expressions of the social presence were intensively associated with students’ performance during their online group discussions (Guo et al., 2021). These results were partly in line with the findings that the social presence has a strong positive relationship with students’ perceived learning and satisfaction (Richardson et al., 2017).

The CoI framework has been widely discussed in the MOOC (massive open online course) context and it was found to be a helpful instrument for designing and delivering the MOOCs (e.g., Patwardhan et al., 2020; Li et al., 2022). For example, the instructors’ facilitation of the teaching presence was very helpful for shaping learners’ attitudes and building a positive learning atmosphere (Watson et al., 2017; Patwardhan et al., 2020); and the instructor played a positive mediating role in the group’s socio-cognitive construction of knowledge and improving learners’ achievements (Liu et al., 2021). Therefore, the CoI was adopted to guide this current study.

## Research questions

The purpose of this study was to examine the significant differences in Chinese college students’ learning perceptions and outcomes between the “quasi smart” and smart classrooms under the perspective of the CoI framework. Specifically, the following three research questions were asked: (a) are there significant differences in students’ perceptions of the teaching, cognitive, and social presences between the “quasi smart” and smart classrooms? (b) Are there significant differences in students’ learning outcomes between the “quasi smart” and smart classrooms? And (c) what are the pros and cons of these two different classroom environments?

## Methodology

### The research design

**FIGURE 2:**
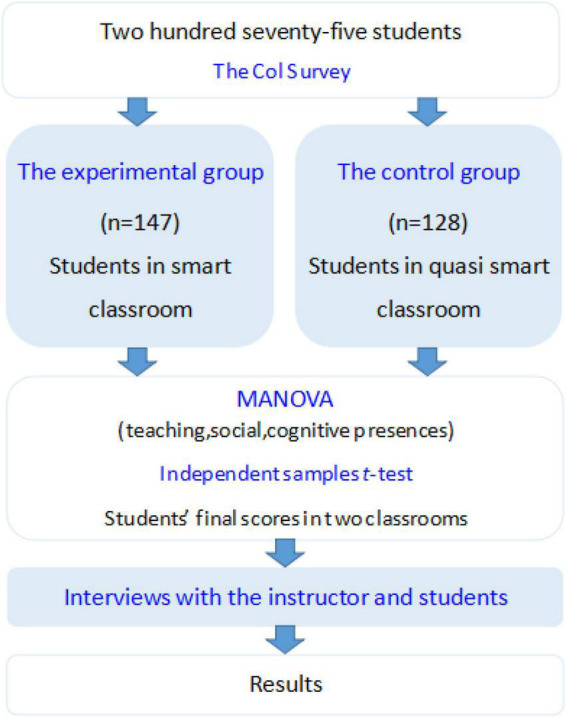
Research design flowchart.

The flowchart of the quasi-experimental research was shown in Figure 2. The participants of this study included 275 students, with 147 and 128 students in the experimental and control groups, respectively. Using the CoI survey, the learning perceptions of both student groups were measured. The MANOVA was first conducted to examine the significant perception difference between the two groups; the independent samples *t*-test was then performed to investigate the significant score difference between the two groups. In addition, interviews with the instructor and students were conducted to examine their environment preferences.

### Research context and participants

The participants of this study included 275 freshmen students who took the “college physics” course in the spring of 2022 at a four-year university in central China. This was a hybrid course. For the face-to-face section, students were placed in either a smart classroom or a “quasi smart” classroom. Among the 275 freshmen students, 147 students were placed in the smart classroom, and 128 students in the “quasi smart” classroom. For the online section, students in both classrooms were using Chaoxing, a popular online learning platform in Chinese higher education. It is important to mention that these two classes were taught by the same professor.

### Instrument and data collection procedures

The instrument for data collection was a five-point Likert survey ranging from 1 (strongly disagree) to 5 (strongly agree). It consisted of 26 items which were selected from the previous literature (Arbaugh et al., 2008; Shea and Bidjerano, 2009) to measure participants’ social (nine items), cognitive (seven items), and teaching (ten items) presences in the hybrid course in different classroom environments (see Table 1). Survey data collection was conducted online with the assistance of the teaching assistant. Further, all students’ final course marks were obtained. The researchers provided all the participants with information of the study and consent forms; they all understood that their participation was totally voluntary and their responses were strictly confidential.

**TABLE 1:** A description of the 26 items with factor loadings.

Item	Description	Factor 1	Factor 2	Factor 3
TP1	The instructor clearly communicated important course topics.	0.802		
TP2	The instructor clearly communicated important course goals.	0.817		
TP3	The instructor provided clear instructions on how to participate in course learning activities.	0.688		
TP4	The instructor clearly communicated important due dates/time frames for learning activities.	0.859		
TP5	The instructor was helpful in identifying areas of agreement and disagreement on course topics that helped me to learn.	0.752		
TP6	The instructor was helpful in guiding the class toward understanding course topics in a way that helped me clarify my thinking.	0.769		
TP7	The instructor helped keep the course participants on task in a way that helped me to learn.	0.670		
TP8	The instructors encouraged course participants to explore new concepts in this course.	0.691		
TP9	Instructor actions reinforced the development of a sense of community among course participants.	0.600		
TP10	The instructor provided feedback in a timely fashion.	0.617		
SP1	Getting to know other course participants gave me a sense of belonging in the course.		0.755	
SP2	I was able to form distinct impressions of some course participants.		0.496	
SP3	Online or web-based communication is an excellent medium for social interaction.		0.806	
SP4	I felt comfortable conversing through the online medium.		0.701	
SP5	I felt comfortable participating in the course discussions.		0.525	
SP6	I felt comfortable interacting with other course participants.		0.823	
SP7	I felt comfortable disagreeing with other course participants while still maintaining a sense of trust.		0.656	
SP8	I felt that my point of view was acknowledged by other course participants.		0.756	
SP9	Online discussions help me to develop a sense of collaboration.		0.732	
CP1	Problems posed increased my interest in course issues.			0.862
CP2	Course activities piqued my curiosity.			0.791
CP3	I felt motivated to explore content related questions.			0.777
CP4	Brainstorming and finding relevant information helped me resolve content related questions.			0.468
CP5	Online discussions were valuable in helping me appreciate different perspectives			0.497
CP6	Learning activities helped me construct explanations/solutions.			0.697
CP7	Reflection on course content and discussions helped me understand fundamental concepts in this class.			0.567

TP, the teaching presence; SP, the social presence; CP, the cognitive presence. Moreover, as shown in Table 2, the CoI survey instrument had been shown to be highly reliable, with an alpha reliability coefficient of 0.95. Further, the ten items for the teaching presence, the nine items for the social presence, and the seven items for the cognitive presence had also been shown to be very reliable, with alpha reliability coefficients of 0.94,0.90, and 0.90, respectively.

**TABLE 2:** Cronbach’s alpha and descriptive statistics of each latent variable.

Latent variable	Item	Alpha	Mean	Standard deviation
TP	TP1-TP10	0.94	4.39	0.51
SP	SP1-SP9	0.90	4.08.	0.55
CP	CP1-CP7	0.90	4.15.	0.59

TP, the teaching presence; SP, the social presence; CP, the cognitive presence.

In addition, a follow-up focus group interview with ten randomly selected participants and a semi-structured interview with the course professor were conducted. Questions about the pros and cons of the “quasi-smart” and smart classroom environments were asked during these interviews. The purpose of these interviews was to answer the last research question. To elicit more in-depth information from the interviewees, the interviews were conducted in Chinese. Furthermore, to ensure the reliability, integrity, and validity of the interview data, the interviews were transcribed and then translated into English by the two joint first authors.

### Data analysis methods

Using IBM SPSS, the survey data were analyzed at the following levels. First, the construct validity and internal consistency reliability of the five-point Likert survey were examined. The exploratory factor analysis (EFA) was performed to identify the three latent constructs as originally hypothesized in the CoI framework. After the correct number of latent constructs was identified, the internal consistency reliability of CoI survey was then calculated.

Further, a one factor multivariate ANOVA analysis (MANOVA) and an independent samples *t*-test were conducted. The purpose of the MANOVA was to examine the significant differences in participants’ perceptions of the teaching, cognitive, and social presences between the “quasi smart” and smart classrooms. The purpose of the independent samples *t*-test was to investigate the significant differences in students’ learning outcomes between the “quasi smart” and smart classrooms.

Finally, the interview data were coded and analyzed by the two joint first authors who have rich experience in qualitative data analysis. Specifically, responses under each interview question were color-coded and sorted into different categories by two of them independently, and then organized collaboratively based on content; and finally, conceptually similar responses were discussed, grouped together, and then categorized according to the recurring themes. To enhance its validity, direct quotes from the interviewees were also incorporated (Creswell, 2014).

## Results

### The construct validity and internal consistency reliability of the community of inquiry survey

A principal component with promax rotation EFA was conducted to examine the construct validity of the CoI survey. The Kaiser-Meyer-Olkin (KMO) measure of sampling adequacy was 0.94. Eigenvalues for three factors were >1; further, the scree plot suggested a three-factor model which explained 63.5% of the total variance (see Table 1). All items had moderate to high loadings (>0.40) on the three common factors.

As shown in Table 1, ten items (items TP1 through TP10) had moderate to high loadings on the first common factor representing the teaching presence; nine items (items SP1 through SP9) had moderate to high loadings on the second common factor representing the social presence; and seven items (items CP1 through CP7) had moderate to high loadings on the third common factor representing the cognitive presence. These results showed that the CoI survey demonstrated good construct validity.

### The one-factor MANOVA results

A one-factor MANOVA was conducted to examine the significant differences in Chinese college students’ perceptions of the teaching, cognitive, and social presences within the CoI framework between the “quasi smart” and smart classrooms. The results are presented in Table 3.

**TABLE 3:** The MANOVA results comparing “quasi smart” and smart classroom environment differences.

Multivariate tests					Tests of between-subjects effects					
Test	Value	*F*	Sig.	Effect size*	Variable	Quasi smart (Mean)	Smart (Mean)	*F*	Sig.	Effect size*
Pillai’s trace	0.44	71.80	0.000	0.44	TP	4.06	4.69	116.54	0.000	0.38
Wilks’ lambda	0.56	71.80	0.000	0.44	SP	3.76	4.36	113.81	0.000	0.29
Hotelling’s trace	0.80	71.80	0.000	0.44	CP	3.78	4.48	145.83	0.000	0.35
Roy’s largest root	0.80	71.80	0.000	0.44						

TP, the teaching presence; SP, the social presence; CP, the cognitive presence.

*Partial Eta Squared.

As shown in Table 3, the four most commonly reported statistical indicators converged to the same equivalent *F*-value. Also the significant *p*-value of less than 0.01 (effect size = 0.44) indicated individual between-group differences. Follow-up univariate ANOVAs indicated that there were significant differences for all three dependent variables of the teaching (*p* < 0.01, effect size = 0.38), social (*p* < 0.01, effect size = 0.29), and cognitive presences (*p* < 0.01, effect size = 0.35). Specifically, the students’ perception of the teaching presence were significantly higher in the smart classroom (mean = 4.69) than in the “quasi smart” classroom(mean = 4.06) (*p* < 0.01, effect size = 0.38);similarly, their perception of the social presence were significantly higher in the smart classroom (mean = 4.36) than in the “quasi smart” classroom (mean = 3.76) (*p* < 0.01, effect size = 0.29); finally, their perception of the cognitive presence were also significantly higher in the smart classroom (mean = 4.48) than in the “quasi smart” classroom (mean = 3.78) (*p* < 0.01, effect size = 0.35).

### The independent samples *t*-test results

In order to examine significant differences in students’ learning outcomes between the “quasi smart” and smart classrooms, an independent samples *t*-test was conducted. The results are presented in Table 4.

**TABLE 4:** The independent samples *t*-test results.

Dependent variable	Grouping variable	*N*	Mean	SD	*t-*value	DF	*p*	Hedges’ g*
Course marks	“Quasi smart”	128	61.40	13.12	2.55	241.05	0.011	0.31
	Smart	147	65.08	10.39				

*The effect size was calculated for the meaningful differences between the two groups. It is important to mention that Cohen’s d is the appropriate effect size measure for the independent samples *t*-tests if the two groups have similar standard deviations and are of the same size. Hedge’s g is a modified Cohen’s d or an alternative where there are different sample sizes for the two groups.

As shown in Table 4, significant differences were found between the students in the “quasi smart” classroom and the students in the smart classroom in terms of their learning outcomes. Specifically, students in the smart classroom (mean = 65.08) achieved significantly higher course marks than those in the “quasi smart” classroom (mean = 61.40) (*p* < 0.05, effect size = 0.31).

### The focus group and semi-structured interview results

The follow-up focus group interview with the ten randomly selected students and semi-structured interview with the course instructor generated the following findings (see Table 5).

**TABLE 5:** A summary of the interview results.

Interview questions	Major themes	
	Student	Instructor
(a) What are the pros of the smart classroom?	(a) Physical environment (b) Accessibility of resources and availability of supporting tools (c) Convenience of learning	(a) Technical support and guidance (b) Teaching methods (c) Student engagement and interaction
(b) What are the cons of the smart classroom?		
(c) What are the pros of the “quasi smart” classroom?		
(d) What are the cons of the “quasi smart” classroom?		

#### Findings of focus group interview with selected participants

The student participants commented on the pros and cons of these two classroom learning environments (i.e., the “quasi smart” and smart classrooms) from the following three aspects: (a) physical environment, (b) accessibility of resources and availability of supporting tools, and (c) convenience of learning (see Table 5).

First, the ten student participants commented positively on the physical environment (e.g., adequate number of sockets, good air conditioning, and aesthetic and feasible tables and chairs) in the smart classroom. Also, “*the flexible desks and chairs in the smart classroom are convenient for group discussions and learning cooperation in smart classroom*,” as mentioned by one participant. By contrast, as many of them mentioned that the tables and chairs are fixed in the “quasi smart” classroom, and it is not convenient to organize group activities in the “quasi smart” classroom.

Second, the student participants argued that the educational technology matters. The accessibility of intelligent devices in the smart classroom could increase the chances of self-learning; and “*the intelligent classroom with automatic screen recording can be played back for us to check the missing content at any time*… *the technology supports our learning before, during, and after class.*, “…*especially there are computers and tablets for us to search information conveniently; and we could try our experiments immediately when we have new ideas in the smart classroom*… *however, the ‘quasi smart’ classroom does not have these features*,” as argued by one participant.

Finally, the student participant made the following positive comments on the convenience of learning in the smart classroom. “*Multi-screen display is good for showing results and receiving information in the smart classroom*;” “*everyone can see what is on the screen*;” “*it is the same wherever you sit*;” “*students can easily interact with each other*” and “*it is good for cooperative learning*.” However, in the “quasi smart” classroom, the students did usually lack such convenience of learning, as agreed by all of the student participants.

According to students’ perspective, the pros and cons of these two different classroom environments are summarized in Table 6.

**TABLE 6:** A summary of focus group interview results.

Students	The pros and cons
Physical environment	The smart classroom is better than the “quasi smart” classroom.
Accessibility of resources and availability of supporting tools	The smart classroom is better than the “quasi smart” classroom.
Convenience of learning	The smart classroom is better than the “quasi smart” classroom.

#### Findings of semi-structured interview with the instructor

Unlike the student participants, the course instructor commented the pros and cons of these two classroom learning environments from different three aspects: (a) technical support and guidance, (b) teaching methods, and (c) student engagement and interaction (see Table 5).

First, the instructor commented that “…*the multi-screen display in the smart classroom is conducive to me walking around the class at any time to explain, and answer questions*… *it shortens the distance between me and my students*… *it enhances the interaction between me and my students*.” In the quasi smart classroom, however, “*there is only one screen in the front of the classroom, so my walking space is limited. moreover, there is a space distance between the instructor[me] and students, which can naturally separate me from my students*.”

Second, the instructor commented that “*the mode of teaching in the smart classroom is basically the same as in quasi smart classroom, with no significant change*.” He continued to explain that “… *many students enrolled in this course [i.e., the college physics course]*…*classroom interaction can adopt the way of asking questions and casting screens through the learning APP or the classroom response system and all students can participate in*.”

Finally, the instructor mentioned that students in the smart classroom are more learning autonomous and feeling more comfortable than in the “quasi smart” classroom. He further commented that “*they [students] can search information at any time, explore knowledge independently, and communicate with classmates freely in the smart classroom in contrast to the “quasi smart” classroom*.”

According to instructor’s perspective, the pros and cons of these two different classroom environments are summarized in Table 7.

**TABLE 7:** A summary of the semi-structured interview results.

Instructor	Pros and cons
Technical support and guidance	The smart classroom is better than the “quasi smart” classroom.
Teaching methods	The smart classroom is the same as the “quasi smart” classroom.
Student engagement and interaction	The smart classroom is better than the “quasi smart” classroom.

## Discussion and conclusions

This study examined the significant differences in Chinese college students’ learning perceptions and outcomes between the “quasi smart” and smart classrooms under the perspective of the CoI framework. The findings of this study were significant. Specifically, the students’ perception of the teaching presence were significantly higher in the smart classroom than in the “quasi smart” classroom; similarly, their perception of the social presence were significantly higher in the smart classroom than in the “quasi smart” classroom; further, their perception of the cognitive presence were also significantly higher in the smart classroom than in the “quasi smart” classroom; finally, students in the smart classroom achieved significantly higher course marks than those in the “quasi smart” classroom.

The results confirmed the difference of the teaching, social, and cognitive presences existed significantly between the smart and quasi smart classrooms. Previous research found that the profound and meaningful online learning was caused by the interaction of such three elements as the teaching, social, and cognitive presences perceived by students in the learning community (Garrison et al., 2010; Shea and Bidjerano, 2013; Castellanos-Reyes, 2020; Patwardhan et al., 2020). Smart terminals and mobile learning tools and platforms to support communication between instructor and students were used to facilitate interaction (MacLeod et al., 2018), which might be the fundamental difference distinguishing the smart from the quasi smart classrooms. Significantly more student-initiated actions were triggered in the smart classroom, which indicated that the students’ dominant position was significantly strengthened in the smart classroom (Zhan et al., 2021). Students’ autonomous learning was enhanced in the smart classroom due to the availability of the Internet access and intelligent devices, by which students had more chances to apply their knowledge collaboratively. When students were applying their knowledge to real-life problems collaboratively, they felt satisfied with the learning experience (Strelan et al., 2020).

Previous study showed that the effective interaction such as the interpersonal interaction between the instructor and students, peer to peer interaction, human–tech interaction supporting instructor’s presentation and management were enhanced in the smart classroom, and the classroom atmosphere was also more harmonious (Yu et al., 2022). Intelligent devices provide more opportunities for the instructor to provide timely and effective guidance to students, and promote individualized and differentiated communication between students and the instructor in the smart classroom.

Furthermore, instructors in the smart classroom could more accurately and quickly analyze students’ learning status through the smart terminals and learning analytics technology (Zhang et al., 2019), accordingly, to adjust the teaching process. Moreover, the structured learning materials and students’ use log data recorded of the resource platform in the smart classroom help to illustrate students’ learning status (Zhai et al., 2016).

In addition, students liked the smart classroom better than the quasi smart classroom because the atmosphere in the smart classroom was more harmonious, which could explain why the social presence in the smart classroom was better than in the quasi smart classroom. The social presence was beneficial for the cognitive presence through motivating the learners, increasing the active community atmosphere, promoting learners’ engagement, and facilitating interaction (e.g., Zhan and Hu, 2013). In addition, researchers put forward that the social presence can overcome the potential negative reactions through enhancing stronger peer connections, reducing feelings of isolation and intensifying feelings of psychological connection and community; and students with high perceptions of the social presence tend to have high perception of learning and satisfaction with their course instructors (e.g., Richardson et al., 2017; Patwardhan et al., 2020; Guo et al., 2021). These might be the reason why the three presences significantly higher in the smart classroom than in the quasi smart classroom.

Finally, previous studies have shown that the classroom interaction could affect the classroom atmosphere, students’ behavior, and the level of engagement (Kaufmann and Vallade, 2020), which could explain why students commented more positively on the smart classroom than the “quasi smart” classroom. In addition, many student participants mentioned that the physical environment was much more comfortable in the smart classroom than in the “quasi smart” classroom, e.g., there are blue walls, flexible desks and chairs, screens around. Since there is only one display screen in the “quasi smart” classroom, class content could be insufficiently or separately displayed, whereas this problem could be solved in the smart classroom, where there are multiple display screens. Previous research has indicated that multi-screen display could reduce students’ cognitive load and improve their academic performance (Cheng et al., 2015).

Furthermore, no steps between lecture desk and students desks. The instructor also stated that no step between the lecture desk and students’ desks is convenient for him to walk around in the classroom; it also bring him much closer to his students for emotional support; it guarantees much more interaction between him and his students; and makes the learning atmosphere in the smart classroom much more harmonious and joyful.

## Data availability statement

The raw data supporting the conclusions of this article will be made available by the authors, without undue reservation.

## Ethics statement

The studies involving human participants were reviewed and approved by the Research Ethic Review Committee of the Evidence-based Research Center for Educational Assessment at Jiangsu University. The patients/participants provided their written informed consent to participate in this study.

## Author contributions

All authors listed have made a substantial, direct, and intellectual contribution to the work, and approved it for publication.
